# Lotions in Porrigo Decalvans and Other Diseases Causing Falling off of the Hair

**Published:** 1846-08

**Authors:** 


*Lotions in Porrigo decalvans and other diseases causing falling off of the hair.*—Mr. Wilson advises that persons with short hair should immerse the head in cold water morning and night, and after drying and well brushing it, to moisten the roots with one of the following lotions. In females with long hair the cold washing may be dispensed with. The lotions are three in number:

No. 1. Vinegar of cantharides, half an ounce.
Eau de Cologne, one ounce.
Rose water, one ounce. Mix.

2. Eau de Cologne, two ounces.
Tincture of cantharides, half an ounce.
Oil of nutmegs, half a drachm.
Oil of lavender, ten drops. Mix.

3- Mezereon bark, one ounce.
Horseradish root, one ounce.
Boiled distilled vinegar, half a pint.

The infusion to stand for a week aud then to be strained. If the hair becomes dry and harsh after using these lotions, a small qnantity of pomatum may be rubbed in.—*Practical Treatise on Healthy Skin, &c.* P. 345.

